# Neurodevelopmental effect of intracranial hemorrhage observed in hypoxic ischemic brain injury in hypothermia-treated asphyxiated neonates - an MRI study

**DOI:** 10.1186/s12887-019-1777-z

**Published:** 2019-11-12

**Authors:** Andrea Lakatos, Márton Kolossváry, Miklós Szabó, Ágnes Jermendy, Hajnalka Barta, Gyula Gyebnár, Gábor Rudas, Lajos R. Kozák

**Affiliations:** 1 Department of Neuroradiology, Medical Imaging Centre, Semmelweis University, Balassa u. 6, Budapest, 1083 Hungary; 2 MTA-SE “Lendület” Cardiovascular Imaging Research Group, Semmelweis University, Városmajor u. 68, Budapest, 1122 Hungary; 3 First Department of Paediatrics, Semmelweis University, Bókay u. 53-54, Budapest, 1083 Hungary

**Keywords:** Asphyxia neonatorum, Brain hypoxia ischemia, Brain hemorrhage, Magnetic resonance imaging, infant development

## Abstract

**Background:**

Identification of early signs of hypoxic ischemic encephalopathy (HIE) with magnetic resonance imaging (MRI) has proven of prognostic significance. Yet, the importance of intracranial hemorrhage (ICH), being present concomitantly had not been investigated yet, despite the known influence of hypothermia on hemostasis. We aimed to determine whether presence of ICH on MRI alongside the signs of HIE have an impact on prognosis in neonates with the clinical diagnosis of HIE.

**Methods:**

A retrospective study of consecutively sampled 108 asphyxiated term infants admitted to a tertiary neonatal intensive care unit (between 2007 and 2016), treated with whole body hypothermia and having brain MRI within 1 week of life was conducted. Presence or absence of HIE signs on MRI (basal ganglia-thalamus, watershed pattern and total brain injury) and on MR spectroscopy (lactate peak with decreased normal metabolites measured by Lac/NAA ratio) and/or of the five major types of ICH were recorded. Neurodevelopmental outcome was measured with Bayley Scales of Infant Development-II (BSID-II) test. Death or abnormal neurodevelopment (BSID-II score < 85) was defined as poor outcome in Chi-square test. Multivariate logistic regression analysis was performed on survivors.

**Results:**

MRI and MR-spectroscopy (MRS) signs of HIE were present in 72% (*n = 78*). 36% (*n = 39*) of neonates had ICH, being mainly small in size. Chi-square test showed a relationship between neurodevelopmental outcome and initial MRI. Unadjusted logistic regression showed that neonates presenting MRI and MRS signs of HIE have 6.23 times higher odds for delayed mental development *(OR = 6.2292; CI95% = [1.2642; 30.6934], p = 0.0246)*, than infants without imaging alterations*;* with no ICH effect on outcome. Adjustment for clinical and imaging parameters did not change the pattern of results, i.e. HIE remained an independent risk factor for delayed neurodevelopment *(OR = 6.2496; CI95% = [1.2018; 32.4983], p = 0.0294)*, while ICH remained to have no significant effect.

**Conclusion:**

HIE related MRI abnormalities proved to be important prognostic factors of poor outcome in cooled asphyxiated infants when present, suggesting that early MRI with MRS is beneficial for prognostication. Interestingly, ICHs present in about one third of all cases had no significant effect on neurodevelopmental outcome, despite the known hemostasis altering effects of hypothermia.

## Background

Hypoxic ischemic encephalopathy (HIE) secondary to perinatal asphyxia is the most common cause of neonatal encephalopathy (NE) [1]. In term or near-term infants (≥36 gestational weeks) with clinical signs of moderate to severe HIE, the current standard is therapeutic whole body hypothermia (33.5 ± 0.2 °C core temperature) for 72 h started within 6 h of life decreasing mortality and morbidity [2, 3], and improving neurologic outcome in survivors [4]. A meta-analysis of several major randomized trials including the National Institute of Child Health and Human Development (NICHD) study [3] and the Total Body Hypothermia (TOBY) trial [4] has demonstrated a reduction in death and neurological impairment at 18 months following hypothermia [5]. The results of the Infant Cooling Evaluation Collaboration (ICE) trial, a multicenter, international, randomized controlled trial shown the reduction of the risk of death and major sensorineural disability at 2 years of age and concluded that whole-body hypothermia is a safe and effective neuroprotective method with minimal adverse effects [6, 7]. However hypothermia treatment also has some consequences that the clinicians need to be aware of during treatment. A Cochrane Collaboration review on the safety of therapeutic hypothermia has shown thrombocytopenia and hypotension to be the major side effects of this therapeutic approach [8]. As cooling may result in decreased platelet counts, it may lead to prolongation of coagulation carrying a potentially higher risk for intracranial hemorrhage (ICH) in HIE newborns [9]. Besides, asphyxia very often leads to impaired cerebral autoregulation [10] and asphyxia and hypothermia might also cause fluctuation of cerebral blood flow [11], being possible risk factors for intraventricular hemorrhage (IVH) [12]. Performing magnetic resonance imaging (MRI) in combination with proton magnetic resonance spectroscopy (MRS) is a widely accepted imaging method for quantifying the extent of hypoxic ischemic brain injury, and predicting outcome [13–24]. Involvement of the posterior limb of the internal capsule (PLIC) was found to be associated with abnormal motor outcome [13, 23]. Early death and the most severe motor impairment as cerebral palsy were seen in association with basal ganglia injuries [23]. In a meta-analysis including thirty-two studies and 860 infants with neonatal encephalopathy deep gray matter Lac/NAA was found to be the most accurate quantitative MR biomarker for prediction of neurodevelopmental outcome [22]. Yet, the different types of ICHs visible on MRI in asphyxiated neonates, were not considered in the previous studies, although they may be of additional impact on the long term outcome of neonatal HIE, as they may carry a potential risk of intracranial complication when hypothermic treatment is adopted [9]. The many previous studies emphasizing the prognostic effect of signal abnormalities on conventional sequences, diffusion restriction pattern, and brain MRS either analyzed a relatively small sample size or MRS was performed after the first week of life or was not involved in the evaluation, meaning that those HIE positive infants having only spectroscopic abnormality were not involved in the study population [13, 15, 25–27].

In this retrospective observational study of cooled infants with the clinical diagnosis of HIE, we aimed to investigate whether the coexistence of ICH on early MRI with the imaging signs of HIE (abnormal MRS and/or typical patterns of diffusion restriction) have an impact on the prognosis.

## Methods

### Study design and setting

This was a retrospective observational study conducted at a university-affiliated tertiary care neonatal intensive care unit functioning as a regional cooling center for neonatal HIE (First Department of Pediatrics, Semmelweis University, Budapest, Hungary). Patients admitted between March 2007 and March 2016 were evaluated.

### Study population

**Fig. 1:**
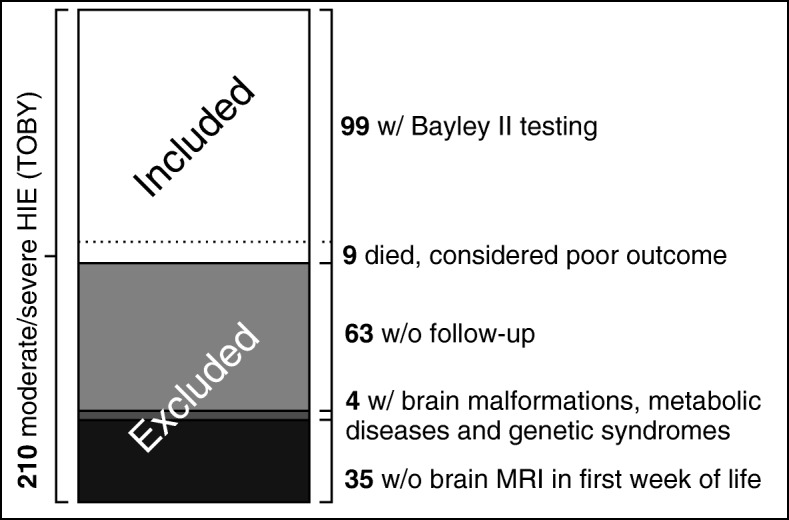
Patient enrollment. Based on the inclusion and exclusion criteria 108 neonates were selected in the study from the initial 210 patients

Patients were selected from 210 consecutive newborn infants (gestational age ≥ 36 weeks). Subjects were included in this study if they were clinically diagnosed as moderate or severe HIE, underwent therapeutic whole body hypothermia and had at least one brain MRI within the first 7 days of life as part of the diagnostic protocol (Fig. 1). As the aim of our study was to investigate ICH present during or early after hypothermia treatment and detect diffusion and spectral changes of HIE known to have a temporal evolution we only considered brain MRI performed during the first week after birth.

Inclusion was based on the following diagnostic criteria of the international TOBY trial (Additional file 1) [4, 8], corresponding to moderate or severe asphyxia: (1) at least of one of the following: continuous need for resuscitation/ventilation at 10 min after birth, OR Apgar score ≤ 5 at five min after birth OR arterial cord pH < 7,0 or base deficit ≥16 mmol/L within 60 min after birth AND (2) altered levels of consciousness (lethargy, stupor, coma) AND hypotonia OR abnormal reflexes or seizures AND (3) abnormal brain background activity registered on amplitude-integrated electroencephalography. Exclusion criteria included: metabolic and/or genetic syndromes, structural brain malformations, lack of neurodevelopmental follow up data. Exception from the neurodevelopmental criterion was made in case of early death (< 28 postnatal days) OR death > 28 days associated with HIE in 9 patients who died before neurodevelopmental testing hence they were considered as poor outcome. Altogether, 108 patients met the criteria and were included in the analysis. There were no significant differences in the main clinical parameters between the included and excluded groups, see Table 1, and the Results section for details.

**Table 1:** Perinatal characteristics of HIE newborns enrolled and excluded from the study. GA: gestational age, BE: base excess

Variable	Included (*n* = 108)	Excluded (*n* = 102)	*p* value
Age at MRI (h) mean ± SD; (range)	65.12 ± 46.34 (4.42–165.67)	–	–
GA (wks) mean ± SD; (range)	38.5 ± 1.51 (36–43)	39.05 ± 1,56 (36–43)	0.1293
Birth weight (g) mean ± SD; (range)	3099.52 ± 447.89 (1490–4300)	3196.78 ± 497.94	0.7357
1-min Apgar score median (range)	2 (0–8)	2 (0–8)	0.4560
5-min Apgar score median (range)	4 (0–9)	5 (0–9)	0.2226
10-min Apgar score median (range)	6 (0–9)	6 (0–9)	0.0698
pH mean ± SD (range)	7.04 ± 0.18 (6.3–7.38)	7.01 ± 0.21 (6.34–7.6)	0.2217
BE (mEq/l) mean ± SD (range)	−16.57 ± 5.94 (− 28–1)	− 16.04 ± 6.55 (− 29–1.7)	0.4985

The primary outcome of the study was to find the possible association between ICH and/or MRI/MRS signs of HIE and neurodevelopmental outcome based on the Bayley Scales of Infant Development II. scale.

The Institutional Ethics Committee (Semmelweis University Regional and Institutional Committee of Scientific and Research Ethics, SE TUKEB 62/2016) and the Medical Research Council Ethics Committee of Hungary (ETT TUKEB 5705–1/2016/EKU) approved this retrospective study.

### Study interventions and exposures

#### Whole-body hypothermia

Whole-body hypothermia treatment was initiated as soon as possible, using a water-filled mattress (Tecotherm©; TecCom, Halle, Germany). The target rectal temperature was between 33 and 34 °C, maintained for 72 h. In the rewarming phase, temperature increase velocity was 0.5 °C/h. All infants were ventilated throughout the cooling and rewarming phase. Knowing that severe hyperoxaemia and severe hypocapnia are associated with adverse outcome in infants with post-asphyxial HIE [28–30] closer monitoring and individualized oxygen supplementation and ventilation was maintained. Initial parameters of mechanical ventilation were set according to the local protocol: synchronized intermittent mandatory ventilation with volume guarantee (target tidal volume 5 ml/kg, respiratory rate and end expiratory pressure set as needed). Taking into consideration of the fact that asphyxiated infants may need lower ventilator settings to maintain normocarbia, our protocol was designed to maintain a pCO2 of 40 to 60 mmHg consistently. Therefore, during the first hours of life, oxygen supplementation and ventilation were rigorously controlled with blood gases routinely taken every 30 to 60 mins, until a steady state was reached, then every 6 h afterwards during the whole extent of hypothermia treatment. This protocol ensured timely adjustment of ventilation parameters to evade sustained excursion from optimal blood gas values.

Continuous morphine (Morphine hydrochloride 10 mg/mL; TEVA Magyarország Zrt., Gödöllő, Hungary) sedation (10 μg/kg BW/h) was started following the loading dose (0.1 mg/kg BW) administered when the cooling was initiated. Phenobarbitone (Gardenal 40 mg; Aventis, Maisons-Alfort, France, 20 mg/kg BW) was given as the first line of anticonvulsant therapy if clinical or electrophysiological seizures were detected. In case of non-controlled seizures, the phenobarbitone loading dose was repeated, or midazolam (Midazolam Torrex 5 mg/ml; Chiesi Pharmaceuticals GmbH, Vienna, Austria) was given in single or repeated doses (0.1 mg/kg BW) or in continuous infusion (0.1 mg/kg BW/h). In some cases, newborns received lidocaine, phenytoin, diazepam or chloral hydrate alternatively, according to the attending clinician’s decision.

#### MR imaging

All MRIs were acquired in the first 7 days of life on a Philips Achieva or Ingenia 3 T scanner (Philips Medical Systems, Best, The Netherlands), with 8-channel SENSE head-coils, at the former MR Research Center of Semmelweis University.^1^ Brain MRI included the following sequences: T1-weighted 3D spoiled gradient echo [TR = 10.88 ms, TE = 5.258 ms, slice thickness = 1 mm, gap =1 mm], axial and coronal T2-weighted turbo spin echo [TR = 4000 ms (axial), 2766 ms (coronal), TE = 100 ms, slice thickness = 3 mm, gap = 4 mm], axial T2*-weighted gradient echo [TR = 561.5 ms, TE = 16.11 ms, slice thickness = 3 mm, gap = 4 mm] OR axial SWI (susceptibility weighted imaging) [TR = 31 ms, TE = 7.2 ms, slice thickness = 2 mm, gap-1 = mm (interleaved slices)] and diffusion-weighted images [b-value = 1000s/mm^2^] with ADC maps, and single-voxel proton MR spectroscopy with PRESS (Point Resolved Spectroscopy) acquisition [TE = 35 and/or 144 and/or 288 ms] obtained from the left thalamus – basal ganglia. Average imaging time was 30 min. A neonatologist was present throughout the procedure. For the time of the examination, the infants were removed from the incubator and received continuous morphine sedation. Heart rate and oxygen saturation were monitored using a Medrad® Veris® MR monitoring system. In case of intubated infants, skilled personnel provided manual ventilation with an AMBU bag throughout the MRI examination.

#### Neurodevelopmental follow-up

Neurodevelopmental outcome was measured by Bayley Scales of Infant Development II tool-kit (BSID-II), performed between 18 and 26 months of age by trained personnel, blinded to the MRI results. Although BSID-II was updated to a 3rd edition during the follow-up period of our study, it was not translated and standardized until 2018, therefore BSID-II test was performed in all our patients. BSID-II comprises two scales including the Mental Developmental Index (MDI) and Psychomotor Developmental Index (PDI). The MDI provides an assessment of memory, problem solving, sensory perception, hand-eye coordination, imitation and early language. The PDI measures gross and fine motor skills [31, 32]. The normal range of PDI and MDI is between 85 and 114, a score lower than 85 suggests mildly delayed development, and a score below 70 represents significantly delayed development. A score greater than or equal to 115 stands for accelerated performance. For the Chi-square test we defined poor outcome as either early death OR mildly to significantly delayed performance (MDI or PDI < 85). All other outcomes were considered as good outcome. A neurodevelopmental outcome score was non-applicable (N/A) when obtaining a reliable measurement was not achievable. Logistic regression analysis was based on individual scores (see below).

#### Data analyses

Data recording and extraction were made using our novel in-house built asphyxia registry database, iSORT (Intelligent structured online reporting tool, Bioscreen Ltd. Debrecen, Hungary) [33]. Trained pediatric radiologist blinded to the clinical history and neurodevelopmental results retrospectively evaluated the imaging signs of HIE and the presence and type of hemorrhage. HIE related abnormality was reported, when a lactate peak and relatively low values of normal metabolites (represented by Lac/NAA height ratios measured on MRS with TE = 144 ms) were present on MR-spectroscopy [34, 35] AND/OR HIE related diffusion restriction or signal abnormalities on T1- and T2 weighted images were present [36–38]. Predominantly basal-ganglia-thalamus pattern was reported when central grey matter nuclei and perirolandic cortex involvement was seen bilaterally with or without associated hippocampal and brain stem involvement and the absence of a normal high-signal intensity of the posterior limb of the internal capsule [37]. Watershed predominant pattern injury was diagnosed when the vascular watershed zones (anterior-middle cerebral artery and posterior-middle cerebral artery) were involved uni- or bilaterally affecting the white matter or in severe cases the overlying cortex as well [37]. In total brain injury widespread involvement of the subcortical white matter and cortex was seen with relative sparing of the immediate periventricular white matter and central grey matter [36]. The predominant (basal ganglia/watershed/total brain injury) patterns of HIE were primarily identified on the DWI and ADC maps on early MRI. As abnormalities observed with diffusion imaging depend on the delay between birth and MRI showing pseudonormalization around the 6th and 7th day after the insult, conventional T1- and T2 weighted sequences were also evaluated. MRI was considered normal in the absence of all the above listed findings. Neonatal intracranial hemorrhage (ICH) was assessed on T2*GRE, SWI and T1-weighted images. The following five subtypes of ICH were distinguished: subdural hemorrhage (SDH), subarachnoid hemorrhage (SAH), germinal matrix hemorrhage (GMH), intraventricular hemorrhage (IVH) and parenchymal hemorrhage (P) [39]. The location, size and the presence/absence of mass-effect were assessed.

Descriptive statistical analysis was carried out; continuous measurements were presented as mean ± SD (min-max) while categorical measurements were presented in numbers and percentages. Parameters of the included and excluded cohorts were compared with Mann-Whitney U test, as some of the data was ordinal (e.g. Apgar, pH, etc.). Patients were categorized into four groups based on the presence or absence of ICH and HIE on MRI. BSID-II scores were registered for every patient, detailed above. Chi-square test was performed to assess whether the observed distributions of PDI and MDI and the MRI signs of HIE and ICH are independent regarding the outcome (null hypothesis) or if there is proof for a relationship between late neurodevelopmental outcome and initial MRI findings (alternative hypothesis). We performed multivariate logistic regression analysis (in Matlab 9.2, The MathWorks Inc. Natick, MA) on survivors to detect possible relationships between poor outcome (BSID-II score < 85) and/or ICH and/or HIE signs on MRI including other covariates as clinical predictors of interest: 5 min Apgar score, baseline pH and age at MRI. One could argue for the inclusion of base excess and/or temperature, however the latter two should not affect the association as they are mostly collinear with other already included parameters, hence they were omitted. The results are provided in terms of odds ratios, and confidence intervals.

## Results

Clinical characteristics of 108 neonates with moderate-to-severe HIE meeting the eligibility criteria and 102 patients excluded from the study are presented in Table 1. Clinical parameters of the included and excluded group of patients showed no significant difference.

The average age at which brain MRI scans were obtained was 65.12 ± 46.34 h. All brain MRIs were performed after passive cooling started during transport or after the initiation of therapeutic hypothermia. Of these 108 patients 9 patients died in the perinatal period (6.67%). None of our patients died between 28 days and the neurodevelopmental follow-up examination. Twelve children had significantly delayed development (MDI and/or PDI < 70). Seven of these patients were diagnosed with cerebral palsy. Twenty-seven children received an MDI and/or PDI score compatible with mildly delayed development (70–84).

**Fig. 2:**
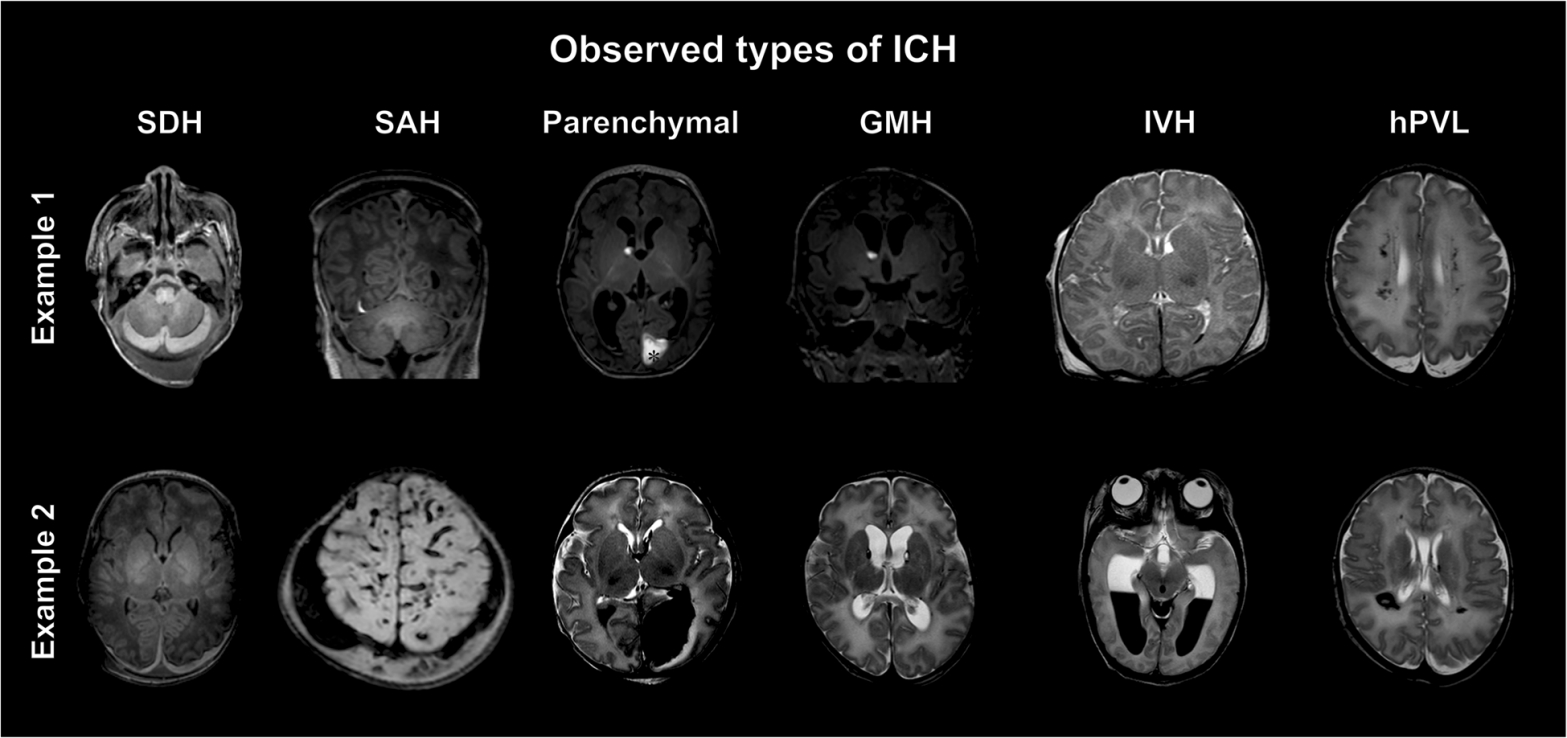
Observed types of ICH. MR images show two sets of examples for intracranial hemorrhages of different types, locations and sizes observed in asphyxiated infants. SDH: subdural hemorrhage, SAH: subarachnoid hemorrhage, GMH: germinal matrix hemorrhage, IVH: intraventricular hemorrhage, hPVL: hemorrhagic periventricular leukomalacia

**Fig. 3:**
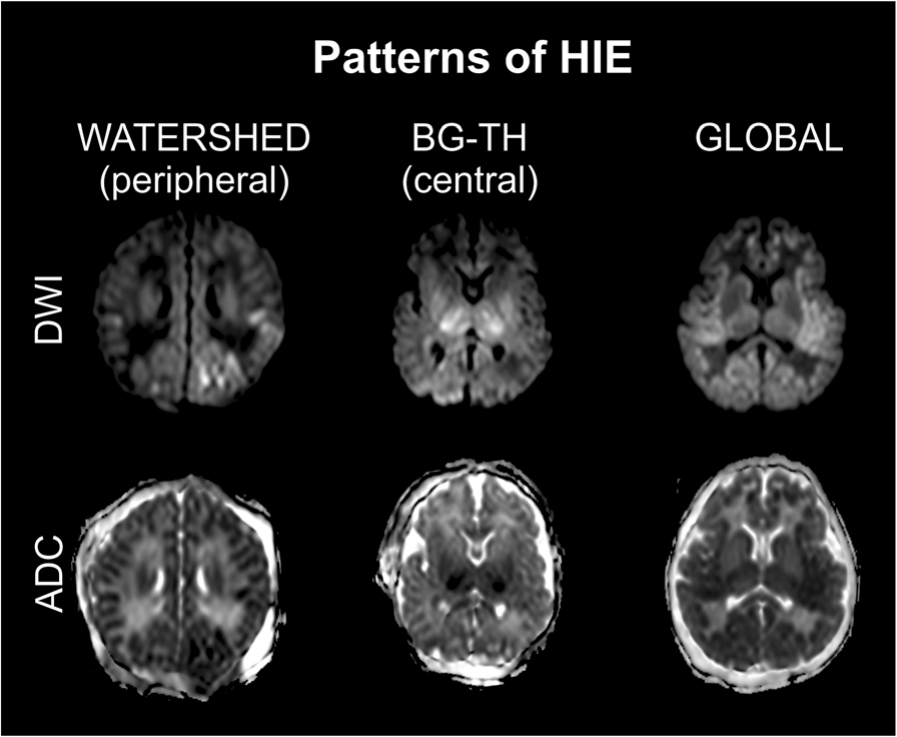
Patterns of HIE. Watershed (peripheral) type of injury, diffusion restriction bilaterally; Basal ganglia – thalamus (central) pattern, restricted diffusion in bilateral thalami and putamina; Global diffusion restriction on DWI and ADC

**Fig. 4:**
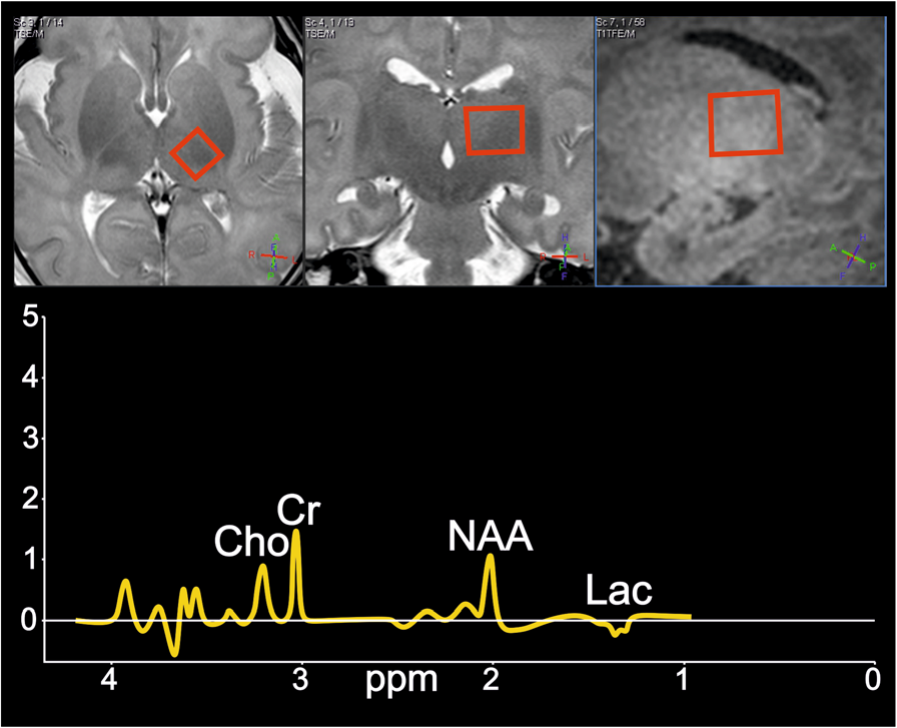
MRS pattern in HIE***.*** Point-Resolved Spectroscopy (PRESS) acquired from the left thalamus (TE = 144 ms) on the 2nd day of life of a term infant shows prominent lactate peak, a characteristic inverse doublet, resonating at 1.3 ppm and decreased N-acetyl-aspartate (NAA), creatine (Cr) and choline (Cho)

The 108 subjects were divided into four groups based on the presence (+) or absence (-) of the MRI signs of ICH (Fig. 2) and HIE (Figs. 3 and 4): Group1: Infants presenting neither the signs of HIE nor ICH (Additional file 2). Group2: Patients without the imaging signs of HIE showing ICH (Additional file 3). Group3: Both HIE and ICH is visible (Additional file 4). Group4: Only HIE signs are present (Additional file 5). Neurodevelopmental outcome results were registered for each patient.

### Group1: HIE−/ICH- cases

Fifteen infants showed no MRI or MRS signs of HIE (although they received cooling based on clinical criteria of moderate to severe HIE) and no ICH. The majority of these patients (80%, *n = 12*) showed normal neurodevelopmental outcome.

### Group2: HIE−/ICH+ cases

Altogether 15 hypothermia treated patients had isolated ICH without the MRI signs of HIE. Eight patients had only one type of extra-axial or parenchymal hemorrhage. The other seven patients had intracranial hemorrhages of multiple types and localizations. The most common ICH types were SDH (*n = 9*) and SAH (*n = 9*), which were small without any mass-effect and caused most probably by traumatic delivery. The extra-axial hemorrhages predominantly occurred in the posterior cranial fossa, along the tentorium cerebelli. IVH was present in three patients, parenchymal hemorrhage in two infants and GMH in one neonate. All fifteen patients with hemorrhage but without the MRI signs of HIE achieved a normal PDI score (≥85) on neurodevelopmental testing. The MDI scores were abnormal in two patients; one of whom had IVH and SAH with multiple foci of GMH besides space occupying cerebral and cerebellar parenchymal hemorrhages leading to hydrocephalus.

### Group3: HIE+/ICH+ cases

Twenty-four patients showed MRI signs of HIE as well as ICH. There were 10 neonates (*41%*) who only had abnormal MRS (without restricted diffusion and T1-, T2- signal abnormality). Four patients (*17%*) were affected by global severe HIE, four neonates (*17%*) showed watershed pattern, and another four (*17%*) presented with basal ganglia-thalamus pattern of injury. In two patients (*8%*) the signs of hemorrhagic PVL were seen. All but one patient (#3.3) had small ICHs, mainly petechial parenchymal hemorrhages in the periventricular white matter (*n = 10*) or non-complicated SAH (*n = 8*), IVH (*n = 9*) and SDH (*n = 7*) supposed to be traumatic in origin. Low MDI and/or PDI scores in keeping with an adverse outcome were present in 50% (*n = 12*) of this patient group.

### Group4: HIE+/ICH- cases

Altogether 54 patients composed the group of HIE signs on MRI without intracranial hemorrhage. Isolated ^1^H-MR-spectroscopy abnormality (without restricted diffusion and T1-, T2- signal abnormality) was present in 19 patients (*35%*). Eight patients (*15%*) were affected by global severe HIE, seven neonates (*13%*) showed watershed pattern, and eighteen infants (*33%*) presented with basal ganglia-thalamus pattern of injury. In two patients (*4%*) the prominent deep medullary veins were suggestive for HIE. Low MDI and/or PDI scores in keeping with an adverse outcome were present in 61% (*n = 33*) of this patient group.

Examples for the observed types of ICH, the main patterns and the typical spectral abnormalities in HIE are presented on Figs. 2, 3 and 4.

### Chi-square test and logistic regression analysis

Data on neurodevelopmental outcome and MRI results of the four groups were summarized in two by four contingency tables (Table 2). Approximately 36% (*n = 39*) of the examined patient population had axial or extra-axial bleeds, mainly parenchymal or subdural hemorrhages. HIE was present in *62*% (*n = 24*) of such patients (all with intracranial hemorrhage), while in the other *38*% (*n = 15*) of the infants with ICH the imaging signs of HIE were completely absent. MRI evidence of HIE was absent in 28% (*n = 30*) of the neonates who were treated with hypothermia. Chi-square test demonstrated significant relationship between ICH and HIE and the outcome measured by PDI (χ^2^ = 13.025*, df = 3, p = 0.0046*) and MDI (χ^2^ = 14.2673, *df = 3, p = 0.0026*).

**Table 2:** Two by four contingency table summarizing results of MRI and MDI, PDI

		HIE−/ICH-	HIE−/ICH+	HIE+/ICH-	HIE+/ICH+	Total
		No. of pts.	No. of pts.	No. of pts.	No. of pts.	
MDI	normal	13	13	24	13	63
	abnormal	2	2	30	10	44
	total	15	15	54	23^a^	107
PDI	normal	12	15	29	14	70
	abnormal	3	0	25	10	38
	total	15	15	54	24	108

Normal MDI ≥85, abnormal MDI < 85. ^a^ MDI was N/A in 1 patient. Lowest expected value = 6.111; χ^2^ = 14.2673; df = 3; *p* = 0.0026. Normal PDI ≥85, abnormal PDI < 85. Lowest expected value = 5.278; χ^2^ = 13.025; df = 3; *p* = 0.0046. (HIE: hypoxic ischemic encephalopathy, ICH: intracranial hemorrhage, MDI: Mental Developmental Index, PDI: Psychomotor Developmental Index)

**Fig. 5:**
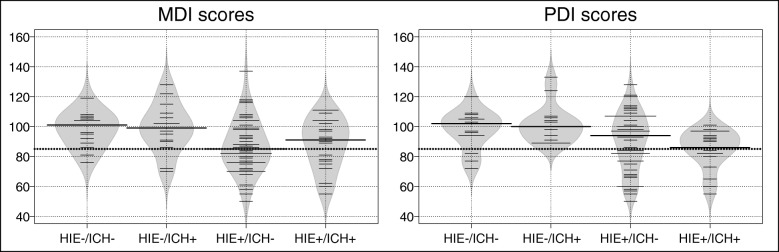
Distribution of neurodevelopmental outcome scores. Bean plots showing the distribution of neurodevelopmental outcome scores (MDI and PDI) in the 4 patient groups. Wide black lines represent the medians, the dotted line at 85 represents the threshold between good and poor outcome

After finding a statistically significant relationship between late neurodevelopmental outcome and HIE and ICH on initial MRI, further analysis using logistic regression was performed in patients whose PDI (*n = 99*) and MDI (*n = 98*) scores were available (*9* infants died early on, one had N/A MDI score). Distribution of neurodevelopmental scores in the four patient groups are presented on bean plots (Fig. 5).

Multivariate logistic regression was used to evaluate the effect of HIE and/or ICH on neurodevelopmental outcome, measured by MDI and PDI scores, adjusting for other clinically relevant parameters including age at MRI, 5 min Apgar score and pH. Table 3. demonstrates the adjusted and unadjusted odds ratios for each variable. Regarding MDI, the unadjusted logistic regression analysis (*χ*^*2*^ *= 11.1, df = 94, p = 0.011*) showed that the presence of MRI signs of HIE had significant negative effect on the outcome (*OR = 6.2292; 95%CI [1.2642; 30.6934]; p = 0.0246)*, meaning neonates who exhibited imaging signs of HIE had six times higher odds of an abnormal MDI score, than those without imaging signs of HIE or ICH. In the same model, neither ICH, nor the simultaneous presence of ICH and HIE had significant modulatory effect (*p > 0.9999* and *p = 0.7121*, respectively). Regarding PDI, the logistic regression analysis (*χ*^*2*^ *= 14.4, df = 95, p = 0.002*) showed that none of the conditions (signs of HIE, ICH, or both) proved to have significant modulatory effect on psychomotor development (*p = 0.2014, p > 0.9999*, and *p > 0.9999*, respectively). After adjusting for confounds, HIE remained an independent risk factor for delayed neurodevelopment with respect to MDI (logistic regression model: *χ*^*2*^ *= 16.000, df = 88, p = 0.014*, effect of HIE: *OR = 6.2496; CI95% = [1.2018; 32.4983], p = 0.0294*), while ICH remained to have no significant effect. Similarly, regarding PDI the adjusted logistic regression model (*χ*^*2*^ *= 16.9, df = 88, p = 0.010*) revealed no modulatory effect of the presence of HIE, ICH, or both (*p = 0.1997, p > 0.9999*, and *p > 0.9999*, respectively). Neither the 5 min Apgar score, nor the pH, nor the time of MRI exam proved to be significant covariates for MDI (*p = 0.1158, p = 0.3415,* and *p = 0.4105*, respectively) or PDI (*p = 0.1675, p = 0.8622*, and *p = 0.5268*, respectively).

**Table 3:** Logistic regression predicting the likelihood of adverse outcome

MDI unadjusted				MDI adjusted		
(model fit: χ^2^ = 11.1, df = 94, *p* = 0.011*)				(model fit: χ^2^ = 16.000, df = 88, *p* = 0.014*)		
	OR	(95% CI)	p	aOR	(95% CI)	p
ICH	NA	NA	0.9999	1.2408	(0.1445–10.6554)	0.8441
HIE	6.2292	(1.2642–30.6934)	0.0246*	6.2496	(1.2018–32.4983)	0.0294*
Time of MRI (h)				0.9954	(0.9847–1.0063)	0.4105
5 min Apgar				0.8259	(0.6507–1.0482)	0.1158
pH				0.3014	(0.0255–3.5682)	0.3415
ICH×HIE	0.6421	(0.0611–6.7517)	0.7121	0.5939	(0.0540–6.5381)	0.6703
PDI unadjusted				PDI adjusted		
(model fit: χ^2^ = 14.4, df = 95, *p* = 0.002*)				(model fit: χ^2^ = 16.9, df = 88, *p* = 0.010*)		
	OR	(95% CI)	p	aOR	(95% CI)	p
ICH	NA	NA	0.9999	NA	NA	0.9999
HIE	2.4828	(0.6152–10.0198)	0.2014	2.5692	(0.6074–10.8666)	0.1997
Time of MRI (h)				0.9963	(0.9851–1.0077)	0.5268
5 min Apgar				0.8443	(0.6639–1.0737)	0.1675
pH				0.8019	(0.0663–9.7067)	0.8622
ICH×HIE	NA	NA	0.9999	NA	NA	0.9999

OR: odds ratio, aOR: adjusted odds ratio, CI confidence interval. Logistic regression model predicting likelihood of adverse outcome on the Bayley Scales of Infant Development II Test, based on the presence of MRI signs of HIE and ICH on early MRI, the time of the MRI examination, the 5 min Apgar score, and the initial pH; asterisks denote significance at level *p* < 0.05

## Discussion

Here, we assessed the rate of ICH in a cohort of patients with clinical signs of HIE, and also considered the effect of ICH on the long term outcome in this patient population. Several studies demonstrate the benefits and compare the accuracy of MRI in identifying HIE and predicting the long term neurodevelopmental outcome [1, 36, 40–42], nevertheless, to our knowledge this was the first study considering the possible relevance of ICH accompanying HIE. Three major randomized controlled trials as the NICHD study [3], the TOBY trial [4] and the ICE trial [6, 7] have demonstrated a reduction in death and neurological impairment after hypothermia treatment. The above trials also included brain MRI, but compared to our study these were relatively late examinations performed after the first week of life in average, assessing the patterns of injury classified by Rutherford mainly on conventional T1- and T2-weighted sequences, and partially on diffusion weighted imaging. The average age on completion of MRI in our study was around the third day of life. The pattern of the injury defined as predominantly basal ganglia or thalamic lesions, watershed predominant injury or global brain insult were classified on diffusion weighted images in most of the cases. MR-spectroscopy was also considered, revealing 29 patients with spectra compatible with HIE and without other imaging signs of hypoxic-ischemic injury.

Published incidence of asymptomatic ICH after vaginal delivery of full-term neonates is approximately 25%, [43] yet symptomatic ICH is much less common, accounting for 4 per 10,000 live births [44]. The rate of ICH in our patient population of cooled asphyxiated infants was higher (36%) than found in literature. The majority of ICHs in our study were small extra-axial or petechial parenchymal hemorrhages localized in the posterior fossa, but there also were multifocal hemorrhages with various patterns of bleeding. The origin of the observed hemorrhages may be related to delivery [45] or to the cooling itself [11, 12], and while the cooling-induced thrombocytopenia [6] may potentially lead to a progression of these bleedings, we have no evidence of progression in our data, partly due to a lack of follow-up imaging. Nevertheless, progression seems to be unlikely given the fact that these ICHs proved to have no significant modulatory effect on the long term mental (MDI) and psychomotor (PDI) developmental outcome.

Early MRI evidence of HIE proved to be a significant predictor of an adverse outcome eventuating more than six times higher odds for abnormal mental development in an asphyxiated child compared to a neonate without the imaging proof of HIE with or without the presence of ICH. Vice versa, in almost all of the cases clinically categorized as HIE (*n* = 30) but lacking MRI evidence of HIE the neurodevelopmental outcome was within normal limits. This is in line with the converging evidence showing the negative outcome associated with the presence of HIE signs on MRI imaging performed after the first week of life [3, 4, 6, 7], and also consistent with the literature regarding early MRI [34].

There are some aspects of the study that can be considered as limitations, the first is the retrospective nature of the data presented. A prospective trial could allow for better controlled patient selection, and could also lead to more consistent data acquisition, nevertheless clinical data obtained in a general setting is equally important [35]. While our data derived from 210 consecutive patients gives a cross sectional overview of the patient population we observed in a 9-year period, it has its own inconsistencies regarding imaging and follow-up, eventually leading to a relatively high rate of exclusion. This may be considered a limitation of our study, nevertheless the lack of significant differences in the basic clinical parameters (e.g. Apgar scores, pH, etc.) between the included and excluded patient cohorts suggests that our sampling is representative of the population categorized as HIE patients based on clinical grounds, i.e. the TOBY criteria [4].

It is important to note, that we excluded those patients whose first MRI was performed after the first week of life, given the main body of HIE-related literature [34, 38, 40] this can be considered as a shortcoming, however our goal was to investigate the early and transient signs of HIE on MRI alongside with the presence of ICH. The fact that our findings on the outcome-modifying effects of HIE signs present on MRI are in line with the literature obtained on later acquired imaging data [3, 4, 6, 7] justifies the appropriateness of our selection criteria.

The relatively wide age range at follow-up developmental testing can also be considered as a potential limitation, however the BSID-II is standardized to be used between 1 and 42 months of age, and the patient’s age is properly taken into consideration for the final scores [32, 46]. Furthermore, one may also consider the use of BSID-II instead of BSID-III a limitation, please note however that the BSID-III was not translated and standardized to Hungarian until 2018; till then the BSID-II was the standard developmental test battery available in Hungarian.

The lack of an objective scaling system to image the severity of HIE may also be considered as limitation. Although, there are several scoring systems available, most of the scales depend on late MRI and conventional images, some of them on the early diffusion pattern alone, yet there is no score including MR spectroscopy in the grading, which could be used in our evaluation [27, 37, 47–49]. The reason for the lack of published MRS grading is that there is very narrow time window for the initiation of the hypothermia (maximum of 6 h from birth) [50, 51], which limits the opportunity of performing an early MRI and there are still many centers where MR-spectroscopy is not included in the imaging protocol [52, 53]. The recent recommendation of the American College of Obstetricians and Gynecologists outlines the importance of early MRI (first 24–96 h of life) for likely timing of the hypoxic-ischemic insult and further follow-up imaging (7–21 days of life) to define the full nature of the abnormalities [54], but does not define or suggest a protocol for brain MRI in an asphyxiated infant.

Finally, one can consider the fact that MR examinations in our study were performed after passive cooling during transport or under or after hypothermic treatment as a limitation of our study, however we do not consider this a clinically relevant limitation as there is published evidence that the predictive value of MRI for subsequent neurological impairment is not affected by previous hypothermia [55]. In fact, MRI is accurate for evaluating the presence and extent of hypoxic brain injury under and after hypothermia [56].

To successfully overcome the above-mentioned limitations more studies on early MRI need to be performed involving more subjects and gathering more imaging data to gain better insight whether there is any interference between hypothermic treatment and imaging that may render the MRI results of asphyxiated infants unremarkable.

## Conclusions

This study attempted to determine the predictive value of intracranial hemorrhage in association with HIE related MR imaging signs in comparison with neurodevelopmental results in cooled asphyxiated infants. Intracranial hemorrhage took a remarkable share affecting approximately one-third of infants treated with hypothermia. Intracranial hemorrhagic complications which were present before or evolve during treatment showed no significant influence on outcome despite the known negative hematological (platelet dysfunction, thrombocytopenia) effects of cooling. Having more than six time higher odds for abnormal neurodevelopmental outcome the presence of HIE related MR-spectroscopy abnormalities and diffusion restriction proved to have important prognostic value. Therefore, the implementation of early MRI in the diagnostic algorithm needs to be considered.

## Supplementary information

**Additional file 1:** Inclusion and exclusion criteria of therapeutic hypothermia in the TOBY trial.

**Additional file 2:** MRI findings and neurodevelopmental outcome in cooled infants with no signs of HIE and ICH on early MRI.

**Additional file 3:** MRI findings and neurodevelopmental outcome in cooled infants with ICH without the imaging signs of HIE.

**Additional file 4:** MRI findings and neurodevelopmental outcome in cooled infants with ICH and the imaging signs of HIE.

**Additional file 5:** MRI findings and neurodevelopmental outcome in cooled infants showing the imaging signs of HIE without ICH.

## Data Availability

The datasets used and analyzed during the current study are available from the corresponding author on request.

## Abbreviations

ADC: Apparent diffusion coefficient
aEEG: amplitude integrated electroencephalography
aOR: adjusted odds ratio
BSID-II: Bayley Scales of Infant Development
DWI: Diffusion weighted imaging
GMH: Germinal matrix hemorrhage
HIE: Hypoxic ischemic encephalopathy
ICE: Infant Cooling Evaluation
ICH: Intracranial hemorrhage
iSORT: Intelligent structured online reporting tool
IVH: Intraventricular hemorrhage
MDI: Mental developmental index
MRI: Magnetic resonance imaging
MRS: Magnetic resonance spectroscopy
NE: Neonatal encephalopathy
NICHD: National Institute of Child Health and Human Development
OR: odds ratio
P: Parenchymal hemorrhage
PDI: Psychomotor developmental index
PLIC: posterior limb of the internal capsule
PRESS: Point RESolved Spectroscopy
SAH: Subarachnoid hemorrhage
SDH: Subdural hemorrhage
SWI: susceptibility weighted imaging
T1: T1 weighted imaging
T2: T2 weighted imaging
TE: Echo time
TOBY: Total body hypothermia for neonatal encephalopathy
TR: Repetition time

## Footnotes

1 The MR Research Center has been reorganized and renamed to Department of Neuroradiology, Medical Imaging Centre, Semmelweis University on August 29, 2019.

## References

[1] Shroff MM, Soares-Fernandes JP, Whyte H, Raybaud C (2010). MR imaging for diagnostic evaluation of encephalopathy in the newborn. Radiographics..

[2] Gluckman PD, Wyatt JS, Azzopardi D, Ballard R, Edwards AD, Ferriero DM (2005). Selective head cooling with mild systemic hypothermia after neonatal encephalopathy: multicentre randomised trial. Lancet..

[3] Shankaran S, Laptook AR, Ehrenkranz RA, Tyson JE, McDonald SA, Donovan EF (2005). Whole-body hypothermia for neonates with hypoxic-ischemic encephalopathy. N Engl J Med.

[4] Azzopardi DV, Strohm B, Edwards AD, Dyet L, Halliday HL, Juszczak E (2009). Moderate hypothermia to treat perinatal asphyxial encephalopathy. N Engl J Med.

[5] Edwards AD, Brocklehurst P, Gunn AJ, Halliday H, Juszczak E, Levene M (2010). Neurological outcomes at 18 months of age after moderate hypothermia for perinatal hypoxic ischaemic encephalopathy: synthesis and meta-analysis of trial data. BMJ..

[6] Jacobs SE, Morley CJ, Inder TE, Stewart MJ, Smith KR, McNamara PJ (2011). Whole-body hypothermia for term and near-term newborns with hypoxic-ischemic encephalopathy: a randomized controlled trial. Arch Pediatr Adolesc Med.

[7] Cheong JL, Coleman L, Hunt RW, Lee KJ, Doyle LW, Inder TE (2012). Prognostic utility of magnetic resonance imaging in neonatal hypoxic-ischemic encephalopathy: substudy of a randomized trial. Arch Pediatr Adolesc Med.

[8] Jacobs SE, Berg M, Hunt R, Tarnow-Mordi WO, Inder TE, Davis PG (2013). Cooling for newborns with hypoxic ischaemic encephalopathy. Cochrane Database Syst Rev.

[9] Forman KR, Diab Y, Wong EC, Baumgart S, Luban NL, Massaro AN (2014). Coagulopathy in newborns with hypoxic ischemic encephalopathy (HIE) treated with therapeutic hypothermia: a retrospective case-control study. BMC Pediatr.

[10] Massaro AN, Jeromin A, Kadom N, Vezina G, Hayes RL, Wang KK (2013). Serum biomarkers of MRI brain injury in neonatal hypoxic ischemic encephalopathy treated with whole-body hypothermia: a pilot study. Pediatr Crit Care Med.

[11] Laptook Abbot R, Corbett Ron J.T (2002). The effects of temperature on hypoxic-ischemic brain injury. Clinics in Perinatology.

[12] Gorelik N, Faingold R, Daneman A, Epelman M (2016). Intraventricular hemorrhage in term neonates with hypoxic-ischemic encephalopathy: a comparison study between neonates treated with and without hypothermia. Quant Imaging Med Surg.

[13] Goergen SK, Ang H, Wong F, Carse EA, Charlton M, Evans R (2014). Early MRI in term infants with perinatal hypoxic-ischaemic brain injury: Interobserver agreement and MRI predictors of outcome at 2 years. Clin Radiol.

[14] Ghei SK, Zan E, Nathan JE, Choudhri A, Tekes A, Huisman TAGM (2014). MR imaging of hypoxic-ischemic injury in term neonates: pearls and pitfalls. Radiographics..

[15] Cavalleri F, Lugli L, Pugliese M, D'Amico R, Todeschini A, Della Casa E (2014). Prognostic value of diffusion-weighted imaging summation scores or apparent diffusion coefficient maps in newborns with hypoxic-ischemic encephalopathy. Pediatr Radiol.

[16] Xu D, Mukherjee P, Barkovich AJ (2013). Pediatric brain injury: can DTI scalars predict functional outcome?. Pediatr Radiol.

[17] Sugiura H, Kouwaki M, Kato T, Ogata T, Sakamoto R, Ieshima A (2013). Magnetic resonance imaging in neonates with total asphyxia. Brain and Development.

[18] Lee ECC, Kwatra NS, Vezina G, Khademian ZP (2013). White matter integrity on fractional anisotropy maps in encephalopathic neonates post hypothermia therapy with normal-appearing MR imaging. Pediatr Radiol.

[19] Harteman JC, Groenendaal F, Toet MC, Benders MJNL, Van Haastert IC, Nievelstein RAJ (2013). Diffusion-weighted imaging changes in cerebral watershed distribution following neonatal encephalopathy are not invariably associated with an adverse outcome. Dev Med Child Neurol.

[20] Gano D, Chau V, Poskitt KJ, Hill A, Roland E, Brant R (2013). Evolution of pattern of injury and quantitative MRI on days 1 and 3 in term newborns with hypoxic-ischemic encephalopathy. Pediatr Res.

[21] Wintermark P, Hansen A, Soul J, Labrecque M, Robertson RL, Warfield SK (2011). Early versus late MRI in asphyxiated newborns treated with hypothermia. Arch Dis Child Fetal Neonatal Ed.

[22] Thayyil S, Chandrasekaran M, Taylor A, Bainbridge A, Cady EB, Chong WK (2010). Cerebral magnetic resonance biomarkers in neonatal encephalopathy: a meta-analysis. Pediatrics..

[23] Rutherford M, Martinez Biarge M, Allsop J, Counsell S, Cowan F (2010). MRI of perinatal brain injury. Pediatr Radiol.

[24] Barkovich AJ, Westmark K, Partridge C, Sola A, Ferriero DM (1995). Perinatal asphyxia: MR findings in the first 10 days. AJNR Am J Neuroradiol.

[25] Azzopardi D, Strohm B, Marlow N, Brocklehurst P, Deierl A, Eddama O (2014). Effects of hypothermia for perinatal asphyxia on childhood outcomes. N Engl J Med.

[26] Ancora G, Testa C, Grandi S, Tonon C, Sbravati F, Savini S (2013). Prognostic value of brain proton MR spectroscopy and diffusion tensor imaging in newborns with hypoxic-ischemic encephalopathy treated by brain cooling. Neuroradiology..

[27] Martinez-Biarge M, Diez-Sebastian J, Kapellou O, Gindner D, Allsop JM, Rutherford MA (2011). Predicting motor outcome and death in term hypoxic-ischemic encephalopathy. Neurology..

[28] Pappas A, Shankaran S, Laptook AR, Langer JC, Bara R, Ehrenkranz RA (2011). Hypocarbia and adverse outcome in neonatal hypoxic-ischemic encephalopathy. J Pediatr.

[29] Lingappan K, Kaiser JR, Srinivasan C, Gunn AJ (2016). Relationship between PCO2 and unfavorable outcome in infants with moderate-to-severe hypoxic ischemic encephalopathy. Pediatr Res.

[30] Klinger G, Beyene J, Shah P, Perlman M (2005). Do hyperoxaemia and hypocapnia add to the risk of brain injury after intrapartum asphyxia?. Arch Dis Child Fetal Neonatal Ed.

[31] Luttikhuizen dos Santos ES, de Kieviet JF, Konigs M, van Elburg RM, Oosterlaan J (2013). Predictive value of the Bayley scales of infant development on development of very preterm/very low birth weight children: a meta-analysis. Early Hum Dev.

[32] Bayley N. Bayley Scales of Infant Development. Second Edition ed: San Antonio,The Psychological Corporation; 1993.

[33] Lakatos A, Kolossvary M, Szabo M, Jermendy A, Bagyura Z, Barsi P (2018). Novel structured MRI reporting system in neonatal hypoxic-ischemic encephalopathy - issues of development and first use experiences. Ideggyogy Sz.

[34] Alderliesten T, de Vries LS, Staats L, van Haastert IC, Weeke L, Benders MJ (2017). MRI and spectroscopy in (near) term neonates with perinatal asphyxia and therapeutic hypothermia. Arch Dis Child Fetal Neonatal Ed.

[35] Barta H, Jermendy A, Kolossvary M, Kozak LR, Lakatos A, Meder U (2018). Prognostic value of early, conventional proton magnetic resonance spectroscopy in cooled asphyxiated infants. BMC Pediatr.

[36] de Vries LS, Groenendaal F (2010). Patterns of neonatal hypoxic-ischaemic brain injury. Neuroradiology..

[37] Barkovich AJ, Hajnal BL, Vigneron D, Sola A, Partridge JC, Allen F (1998). Prediction of neuromotor outcome in perinatal asphyxia: evaluation of MR scoring systems. AJNR Am J Neuroradiol.

[38] Guo L, Wang D, Bo G, Zhang H, Tao W, Shi Y (2016). Early identification of hypoxic-ischemic encephalopathy by combination of magnetic resonance (MR) imaging and proton MR spectroscopy. Exp Ther Med.

[39] Brouwer AJ, Groenendaal F, Koopman C, Nievelstein RJ, Han SK, de Vries LS (2010). Intracranial hemorrhage in full-term newborns: a hospital-based cohort study. Neuroradiology..

[40] Charon V, Proisy M, Bretaudeau G, Bruneau B, Pladys P, Beuchee A (2016). Early MRI in neonatal hypoxic-ischaemic encephalopathy treated with hypothermia: prognostic role at 2-year follow-up. Eur J Radiol.

[41] Chakkarapani E, Poskitt KJ, Miller SP, Zwicker JG, Xu Q, Wong DS (2016). Reliability of early magnetic resonance imaging (MRI) and necessity of repeating MRI in noncooled and cooled infants with neonatal encephalopathy. J Child Neurol.

[42] Charon V, Proisy M, Ferre JC, Bruneau B, Treguier C, Beuchee A (2015). Comparison of early and late MRI in neonatal hypoxic-ischemic encephalopathy using three assessment methods. Pediatr Radiol.

[43] Looney CB, Smith JK, Merck LH, Wolfe HM, Chescheir NC, Hamer RM (2007). Intracranial hemorrhage in asymptomatic neonates: prevalence on MR images and relationship to obstetric and neonatal risk factors. Radiology..

[44] Gupta SN, Kechli AM, Kanamalla US (2009). Intracranial hemorrhage in term newborns: management and outcomes. Pediatr Neurol.

[45] Chaturvedi A, Chaturvedi A, Stanescu AL, Blickman JG, Meyers SP (2018). Mechanical birth-related trauma to the neonate: an imaging perspective. Insights into imaging.

[46] Bos AF (2013). Bayley-II or Bayley-III: what do the scores tell us?. Dev Med Child Neurol.

[47] Liauw L, Palm-Meinders IH, van der Grond J, Leijser LM, le Cessie S, Laan LA (2007). Differentiating normal myelination from hypoxic-ischemic encephalopathy on T1-weighted MR images: a new approach. AJNR Am J Neuroradiol.

[48] Kitamura G, Kido D, Wycliffe N, Jacobson JP, Oyoyo U, Ashwal S (2011). Hypoxic-ischemic injury: utility of susceptibility-weighted imaging. Pediatr Neurol.

[49] Weeke LC, Groenendaal F, Mudigonda K, Blennow M, Lequin MH, Meiners LC (2018). A novel magnetic resonance imaging score predicts neurodevelopmental outcome after perinatal asphyxia and therapeutic hypothermia. J Pediatr.

[50] Colbourne F, Corbett D (1995). Delayed postischemic hypothermia: a six month survival study using behavioral and histological assessments of neuroprotection. J Neurosci.

[51] Taylor DL, Mehmet H, Cady EB, Edwards AD (2002). Improved neuroprotection with hypothermia delayed by 6 hours following cerebral hypoxia-ischemia in the 14-day-old rat. Pediatr Res.

[52] Gunn AJ, Thoresen M (2006). Hypothermic neuroprotection. NeuroRx..

[53] Drury PP, Bennet L, Gunn AJ (2010). Mechanisms of hypothermic neuroprotection. Semin Fetal Neonatal Med.

[54] Executive summary (2014). Neonatal encephalopathy and neurologic outcome, second edition. Report of the American College of Obstetricians and Gynecologists’ task force on neonatal encephalopathy. Obstet Gynecol.

[55] Rutherford M, Ramenghi LA, Edwards AD, Brocklehurst P, Halliday H, Levene M (2010). Assessment of brain tissue injury after moderate hypothermia in neonates with hypoxic-ischaemic encephalopathy: a nested substudy of a randomised controlled trial. The Lancet Neurology.

[56] Boudes E, Tan X, Saint-Martin C, Shevell M, Wintermark P (2015). MRI obtained during versus after hypothermia in asphyxiated newborns. Arch Dis Child Fetal Neonatal Ed.

