# Supplementary material for: Neurodevelopmental effect of intracranial hemorrhage observed in hypoxic ischemic brain injury in hypothermia-treated asphyxiated neonates - an MRI study
Source: BMC Pediatr. 2019 Nov 12;19:430. doi: 10.1186/s12887-019-1777-z (PMC6849254; doi:10.1186/s12887-019-1777-z)
Supplement: Supplementary file 1 — Additional file 1: Inclusion and exclusion criteria of therapeutic hypothermia in the TOBY trial. [file 12887_2019_1777_MOESM1_ESM.docx]

| *Inclusion and Exclusion Criteria of Therapeutic Hypothermia (TOBY trial)* | | |
| --- | --- | --- |
| *INCLUSION CRITERIA:*  The infant will be assessed sequentially by criteria A, B and C listed below. Infants that meet criteria A will be assessed for whether they meet the neurological abnormality entry criteria (B) by trained personnel. Infants that meet criteria A & B will be assessed by aEEG (read by trained personnel). | | |
| ***A.****Infants ≥ 36 completed weeks gestation admitted to the NICU with at least one of the following:* | ***B.****Moderate to severe encephalopathy, consisting of altered state of consciousness (lethargy, stupor or coma) AND at least one of the following:* | ***C.****At least 30 minutes duration of aEEG recording that shows abnormal background aEEG activity or seizures. There must be one of the following:* |
| • Apgar score of ≤ 5 at 10 minutes after birth | • hypotonia | • normal background with some seizure activity |
| • Continued need for resuscitation, including endotracheal or mask ventilation, at 10 minutes after birth | • abnormal reflexes including oculomotor or pupillary abnormalities | • moderately abnormal activity |
| • Acidosis within 60 minutes of birth (defined as any occurrence of umbilical cord, arterial or capillary pH < 7.00) | • absent or weak suck | • suppressed activity |
| • Base Deficit ≥ 16 mmol/L in umbilical cord or any blood sample (arterial, venous or capillary) within 60 minutes of birth | • clinical seizures | • continuous seizure activity |
| *EXCLUSION CRITERIA:* • Infants expected to be > 6 hours of age at the time of randomization  • Major congenital abnormalities, such as diaphragmatic hernia requiring ventilation, or congenital abnormalities suggestive of chromosomal anomaly or other syndromes that include brain dysgenesis. | | |

***Additional file 1*. Inclusion and exclusion criteria of therapeutic hypothermia in the TOBY trial.**
