# Supplementary material for: Neurodevelopmental effect of intracranial hemorrhage observed in hypoxic ischemic brain injury in hypothermia-treated asphyxiated neonates - an MRI study
Source: BMC Pediatr. 2019 Nov 12;19:430. doi: 10.1186/s12887-019-1777-z (PMC6849254; doi:10.1186/s12887-019-1777-z)
Supplement: Supplementary file 2 — Additional file 2: MRI findings and neurodevelopmental outcome in cooled infants with no signs of HIE and ICH on early MRI. [file 12887_2019_1777_MOESM2_ESM.docx]

| *Group1: HIE-/ICH-* | | | |
| --- | --- | --- | --- |
| *No. of pts*. | *Comments, MRI findings* | *MDI* | *PDI* |
| 1.1 | negative | normal, 108 | normal, 105 |
| 1.2 | negative | normal, 95 | normal, 102 |
| 1.3 | negative | normal, 92 | normal, 97 |
| 1.4 | negative | normal, 96 | normal, 96 |
| 1.5 | negative | normal, 104 | normal, 108 |
| 1.6 | negative | normal, 86 | normal, 120 |
| 1.7 | negative | normal, 106 | normal, 109 |
| 1.8 | negative | normal, 89 | normal, 103 |
| 1.9 | negative | normal, 105 | normal, 94 |
| 1.10 | negative | normal, 119 | normal, 94 |
| 1.11 | negative | normal, 107 | normal, 105 |
| 1.12 | negative | normal 101 | normal, 106 |
| 1.13 | negative | abnormal, 76 | abnormal, 77 |
| 1.14 | negative | abnormal, 81 | abnormal, 72 |
| 1.15 | negative | normal, 104 | abnormal, 82 |

***Additional file 2.* MRI findings and neurodevelopmental outcome in cooled infants with no signs of HIE and ICH on early MRI.** Normal MDI & PDI ≥85, abnormal MDI & PDI <85. (SI: signal intensity, WM: white matter, MDI: Mental Developmental Index, PDI: Psychomotor Developmental Index).
