# Supplementary material for: Neurodevelopmental effect of intracranial hemorrhage observed in hypoxic ischemic brain injury in hypothermia-treated asphyxiated neonates - an MRI study
Source: BMC Pediatr. 2019 Nov 12;19:430. doi: 10.1186/s12887-019-1777-z (PMC6849254; doi:10.1186/s12887-019-1777-z)
Supplement: Supplementary file 3 — Additional file 3: MRI findings and neurodevelopmental outcome in cooled infants with ICH without the imaging signs of HIE. [file 12887_2019_1777_MOESM3_ESM.docx]

| *Group2: HIE-/ICH+* | | | | | | | |
| --- | --- | --- | --- | --- | --- | --- | --- |
| *No. of pts.* | *Type of ICH* | *Localization of ICH* | *ICH max. size (mm)* | *ICH mass effect* | *Comments* | *MDI* | *PDI* |
| 2.1 | SDH | B. occipital, B. infratentorial | ≤3 | N |  | normal, 122 | normal, 124 |
| 2.2 | SDH, SAH | SDH: B. occipital, B. infratentorial,  SAH: B. fronto-temporo-parietal | ≤7, ≤1 | N, N |  | normal, 109 | normal, 103 |
| 2.3 | SDH, SAH | SDH: B. occipital, B. parafalcial, B. infratentorial,  SAH: B. occipital | ≤4, ≤1 | N, N |  | normal, 95 | normal, 100 |
| 2.4 | IVH | L. occipital | ≤7 | N |  | normal, 128 | normal, 133 |
| 2.5 | SAH, IVH | SAH: B. occipital, IVH: L. occipital | ≤2, ≤6 | N, N |  | normal, 97 | normal, 107 |
| 2.6 | SDH, SAH | SDH: R. parieto-occipital, B. infratentorial SAH: B. occipital, B. infratent. | ≤2, ≤2 | N, N |  | normal, 90 | normal, 100 |
| 2.7 | GMH1,GMH2, SAH, IVH, P1, P2, Plexus | GMH1: R. caudothalamic,  GMH2: B. mpx. perivent., SAH: L. infratent., IVH: B. occip., P1: L. occip., P2: R. cerebellar, Plexus: R. frontal | ≤6, ≤1, ≤1, ≤2, 15x19x27, 39x33x37, ≤5 | N, N, N, N, Y, Y, N | Hydro- cephalus | abnormal, 72 | normal,  89 |
| 2.8 | SDH, SAH | SDH: B. frontal,  SAH: L. fronto-temporal, B. occipital, B. infratent. | ≤4, ≤1 | N, N |  | normal, 102 | normal,  89 |
| 2.9 | SDH | R. infratentorial | ≤3 | N |  | normal, 91 | normal,  89 |
| 2.10 | SDH | R. infratentorial | ≤2 | N |  | normal, 115 | normal, 104 |
| 2.11 | P | L. occipital | 10x5 | N |  | abnormal, 70 | normal, 100 |
| 2.12 | SDH, SAH | SDH: B. parafalcial, B. infratentorial,  SAH: B. occipital, B. infratentorial | ≤4, ≤1 | N, N |  | normal, 102 | normal,  94 |
| 2.13 | SDH | L. parafalcial, R. infratentorial | ≤2 | N |  | normal, 86 | normal,  91 |
| 2.14 | SAH | L. frontal | ≤1 | N |  | normal, 106 | normal, 106 |
| 2.15 | SAH | R. temporo-occipital | ≤1 | N |  | normal, 99 | normal,  98 |

***Additional file 3*. MRI findings and neurodevelopmental outcome in cooled infants with ICH without the imaging signs of HIE.** Normal MDI & PDI ≥85, abnormal MDI & PDI <85. (SDH: subdural hemorrhage, SAH: subarachnoid hemorrhage, IVH: intraventricular hemorrhage, GMH: germinal matrix hemorrhage, P: parenchymal hemorrhage, L: left side, R: right side, B: bilateral, N: no mass effect, Y: hemorrhage with mass effect, PCA: posterior cerebral artery, MDI: Mental Developmental Index, PDI: Psychomotor Developmental Index).
