# Supplementary material for: Neurodevelopmental effect of intracranial hemorrhage observed in hypoxic ischemic brain injury in hypothermia-treated asphyxiated neonates - an MRI study
Source: BMC Pediatr. 2019 Nov 12;19:430. doi: 10.1186/s12887-019-1777-z (PMC6849254; doi:10.1186/s12887-019-1777-z)
Supplement: Supplementary file 4 — Additional file 4: MRI findings and neurodevelopmental outcome in cooled infants with ICH and the imaging signs of HIE. [file 12887_2019_1777_MOESM4_ESM.docx]

| *Group3: HIE+/ICH+* | | | | | | | | | |
| --- | --- | --- | --- | --- | --- | --- | --- | --- | --- |
| *No. of pts.* | *Type of ICH* | *Localization of ICH* | *ICH max. size (mm)* | *ICH mass effect* | *Pattern of HIE* | *HIE on MRS, Lac/NAA ratio* | *Comments* | *MDI* | *PDI* |
| 3.1 | SAH, IVH, P | SAH: B. fronto-temporo-parietal, IVH: B. occipital horn,  P: B. mpx. fronto-temporo-parietal | ≤1; ≤7; ≤1 | N, N, N | Global | N | Diffuse brain swelling, EXITUS | abnormal | abnormal |
| 3.2 | P | Periventricular by R. occipital horn | ≤1 | N | Hemorrh. PVL | Y,  -0.0416 |  | normal, 89 | abnormal, 82 |
| 3.3 | P | P1: R. occipital,  P2: L. occipital | 21x21x30; 38x26x26 | Y, Y | Watershed (peripheral) | Y,  -0.0385 |  | normal, 93 | normal,  92 |
| 3.4 | P | R. frontal | ≤5 | N | - | Y,  -0.0173 |  | normal, 104 | normal,  93 |
| 3.5 | IVH, GMH, P | IVH: B. occipital, GMH: L. caudothalamic,  P: L. cerebellar | ≤2; ≤1; ≤4 | N, N. N | - | Y,  -0.0121 |  | normal, 102 | normal,  97 |
| 3.6 | IVH | B. occipital | ≤3 | N | - | Y,  -0.1544 |  | abnormal, 78 | abnormal, 82 |
| 3.7 | IVH | B. occipital | ≤2 | N | BG-TH (Central) | Y,  -0.0395 |  | normal, 92 | abnormal, 65 |
| 3.8 | SDH, SAH | SDH: B. occipital, B. infratentorial, SAH: B. occipital, B. infratentorial | ≤3; ≤1 | N,N | - | Y,  -0.0364 | Suspected thrombosis of SSS & transverse sinuses | normal, 109 | normal, 101 |
| 3.9 | IVH | L. occipital | ≤4 | N | Watershed (peripheral) | Y,  -0.0268 |  | normal, 98 | normal,  86 |
| 3.10 | SAH | B. occipital, B. infratentorial | ≤1 | N | - | Y,  -0.0253 |  | normal, 104 | normal,  97 |
| 3.11 | P | B. peritrigonal | ≤1 | N | - | Y,  -0.0688 |  | normal, 91 | normal,  98 |
| 3.12 | IVH | B. occipital | ≤4 | N | - | Y,  -0.9361 |  | abnormal, 62 | abnormal, 80 |
| 3.13 | SDH, IVH | SDH: B. supra-, infratentorial,  IVH: R. occipital | ≤3; ≤2 | N, N | - | Y,  -0.0290 |  | normal, 111 | normal,  91 |
| 3.14 | SDH, SAH | SDH: B. infratentorial,  SAH: B. parietal | ≤4; ≤1 | N, N | BG-TH (Central) | Y,  -0.0118 |  | normal, 97 | normal,  90 |
| 3.15 | SDH, SAH | SDH: B. infratentorial,  SAH: B. occipital | ≤2; ≤1 | N, N | - | Y,  -0.0394 |  | normal, 111 | normal,  94 |
| 3.16 | SAH, IVH | SAH: B. infra-, supratentorial, IVH: B. occipital | ≤1; ≤1 | N, N | Global | Y,  -0.1366 |  | abnormal, 60 | abnormal, 73 |
| 3.17 | SDH, SAH | SDH: B. infra-, supratentorial, SAH: L. parieto-occipital | ≤2; ≤1 | N, N | - | Y,  -0.0271 |  | N/A* | normal, 105 |
| 3.18 | P | R. putamen | 14x5 | N | BG-TH (Central) | Y,  -0.0754 |  | abnormal, 55 | abnormal, 60 |
| 3.19 | SDH, IVH | SDH: B. infra-, supratentorial, IVH: B. occipital | ≤2; ≤6 | N, N | BG-TH (Central) | Y,  -0.0823 |  | abnormal, 75 | abnormal, 55 |
| 3.20 | P | Mpx. B. fronto-parieto-occipital periventricular WM | ≤5 | N | Watershed (peripheral) | Y,  -0.0664 | SSS & transverse sinus thrombosis | abnormal, 77 | normal,  86 |
| 3.21 | P | Mpx. B. hemispheres | ≤2 | N | Global | Y,  -0.1083 | EXITUS | abnormal | abnormal |
| 3.22 | P | Mpx. B. hemispheres, periventricular WM | ≤1 | N | Hemorrh. PVL | Y,  -0.0553 |  | abnormal, 72 | normal,  97 |
| 3.23 | SDH, SAH | SDH: B. supra-, infratentorial, SAH: B. supra-, infratentorial | ≤3; ≤1 | N, N | Watershed (peripheral) | Y,  -0.0595 |  | normal, 90 | normal,  86 |
| 3.24 | P | Mpx. B. cerebellar | ≤5 | N | Global | Y,  -0.1482 |  | abnormal, 81 | abnormal, 84 |

***Additional file 4*. MRI findings and neurodevelopmental outcome in cooled infants with ICH and the imaging signs of HIE.** Normal MDI & PDI ≥85, abnormal MDI & PDI <85. *N/A: non applicable, no reliable measurement was available, Lac/NAA ratio was calculated based on heights of metabolite peaks on MRS acquired with TE=144ms (SDH: subdural hemorrhage, SAH: subarachnoid hemorrhage, IVH: intraventricular hemorrhage, GMH: germinal matrix hemorrhage, P: parenchymal hemorrhage, L: left side, R: right side, B: bilateral, N: no mass effect, Y: hemorrhage with mass effect, WM: white matter, BG-TH: basal ganglia thalamus pattern, PVL: periventricular leukomalacia, SSS: superior sagittal sinus, MDI: Mental Developmental Index, PDI: Psychomotor Developmental Index).
