# Supplementary material for: Neurodevelopmental effect of intracranial hemorrhage observed in hypoxic ischemic brain injury in hypothermia-treated asphyxiated neonates - an MRI study
Source: BMC Pediatr. 2019 Nov 12;19:430. doi: 10.1186/s12887-019-1777-z (PMC6849254; doi:10.1186/s12887-019-1777-z)
Supplement: Supplementary file 5 — Additional file 5: MRI findings and neurodevelopmental outcome in cooled infants showing the imaging signs of HIE without ICH. [file 12887_2019_1777_MOESM5_ESM.docx]

| *Group4: HIE+/ICH-* | | | | | |
| --- | --- | --- | --- | --- | --- |
| *No. of pts.* | *Type of HIE* | *HIE on MRS, Lac/NAA* | *Comments* | *MDI* | *PDI* |
| 4.1 | - | Y,  -0.0297 |  | normal, 106 | normal,  97 |
| 4.2 | - | Y,  -0.0756 |  | normal, 92 | normal,  100 |
| 4.3 | B. prominent deep medullary veins | N |  | abnormal, 58 | abnormal,  77 |
| 4.4 | B. prominent deep medullary veins | Y,  -0.0327 |  | normal, 98 | normal,  112 |
| 4.5 | - | Y,  -0.0271 |  | normal, 101 | normal,  111 |
| 4.6 | BG-TH (Central) | Y,  -0,0867 |  | normal, 86 | abnormal,  84 |
| 4.7 | BG-TH (Central) | Y,  -0.0721 |  | normal, 107 | normal,  91 |
| 4.8 | BG-TH (Central) | Y,  -0.1036 |  | abnormal, 76 | abnormal,  71 |
| 4.9 | BG-TH (Central) | Y,  -0.0793 |  | normal, 93 | normal,  107 |
| 4.10 | BG-TH (Central) | N |  | normal, 98 | normal,  101 |
| 4.11 | - | Y,  -0.0529 |  | normal, 104 | normal,  100 |
| 4.12 | BG-TH (Central) | Y,  -0.1482 |  | abnormal, 55 | abnormal,  50 |
| 4.13 | BG-TH (Central) | N |  | abnormal, 76 | normal,  93 |
| 4.14 | Global | Y,  -0.1543 | EXITUS | abnormal | abnormal |
| 4.15 | Global | Y,  -0.0616 |  | normal, 99 | abnormal,  75 |
| 4.16 | BG-TH (Central) | N |  | normal, 104 | normal,  107 |
| 4.17 | Global | Y,  -0.0867 |  | abnormal, 76 | abnormal,  77 |
| 4.18 | BG-TH (Central) | Y,  -0.2224 |  | abnormal, 77 | abnormal,  58 |
| 4.19 | BG-TH (Central) | N |  | normal, 99 | normal,  107 |
| 4.20 | Watershed (Peripheral) | N |  | normal, 94 | normal,  104 |
| 4.21 | Watershed (Peripheral) | Y,  -0.0816 |  | abnormal, 70 | normal,  93 |
| 4.22 | Global | Y,  -0.1308 | EXITUS | abnormal | abnormal |
| 4.23 | - | Y,  -0.0549 |  | normal, 118 | normal,  128 |
| 4.24 | Watershed (Peripheral) | Y,  -0.0756 |  | abnormal, 82 | normal,  98 |
| 4.25 | Global | Y,  -0.0664 |  | normal, 87 | abnormal,  66 |
| 4.26 | BG-TH (Central) | Y,  -0.0477 |  | abnormal, 68 | normal,  97 |
| 4.27 | - | Y,  -0.1300 | EXITUS | abnormal | abnormal |
| 4.28 | BG-TH (Central) | Y,  -0.0348 |  | normal, 108 | normal,  121 |
| 4.29 | BG-TH (Central) | Y,  -0.0777 | EXITUS | abnormal | abnormal |
| 4.30 | Global | N |  | abnormal, 71 | abnormal,  68 |
| 4.31 | - | Y,  -0.5528 |  | normal, 85 | normal,  94 |
| 4.32 | - | Y,  -0.5951 |  | abnormal, 82 | normal,  94 |
| 4.33 | - | Y,  -0.0268 |  | normal, 90 | normal,  114 |
| 4.34 | Watershed (Peripheral) | Y,  -0.1315 |  | abnormal, 80 | abnormal,  55 |
| 4.35 | - | Y,  -0.0553 |  | normal, 116 | normal,  113 |
| 4.36 | BG-TH (Central) | Y,  -0.6413 | EXITUS | abnormal | abnormal |
| 4.37 | - | Y,  -0.0253 |  | normal, 88 | normal,  98 |
| 4.38 | - | Y,  -0.0384 |  | abnormal, 83 | normal,  94 |
| 4.39 | - | Y,  -0.0775 |  | abnormal, 72 | abnormal,  82 |
| 4.40 | Watershed (Peripheral) | Y,  -0.0629 | embolization | normal, 88 | normal,  97 |
| 4.41 | - | Y,  -0.0635 |  | abnormal, 70 | abnormal,  57 |
| 4.42 | - | Y,  -0.0592 |  | abnormal, 82 | abnormal,  60 |
| 4.43 | - | Y,  -0.0040 |  | normal, 117 | normal,  109 |
| 4.44 | - | Y,  -0.0121 |  | abnormal, 75 | normal,  85 |
| 4.45 | - | Y,  -0.0782 |  | abnormal, 82 | abnormal,  82 |
| 4.46 | - | Y,  -0.0650 |  | abnormal, 84 | abnormal,  84 |
| 4.47 | BG-TH (Central) | Y,  -0.0325 |  | abnormal, 50 | abnormal,  60 |
| 4.48 | Global | N | PVL | abnormal, 79 | abnormal,  67 |
| 4.49 | BG-TH (Central) | Y,  -1.0311 | EXITUS | abnormal | abnormal |
| 4.50 | BG-TH (Central) | Y,  -0.0017 |  | normal, 137 | normal,  107 |
| 4.51 | Watershed (Peripheral) | N |  | abnormal, 70 | abnormal,  82 |
| 4.52 | BG-TH (Central) | Y,  -0.0362 |  | normal, 86 | normal,  120 |
| 4.53 | Watershed (Peripheral) | N |  | abnormal, 61 | normal,  86 |
| 4.54 | Global | Y,  -0.0855 | EXITUS | abnormal | abnormal |

***Additional file 5.* MRI findings and neurodevelopmental outcome in cooled infants showing the imaging signs of HIE without ICH.** Normal MDI & PDI ≥85, abnormal MDI & PDI <85, Lac/NAA ratio was calculated based on heights of metabolite peaks on MRS acquired with TE=144ms (BG-TH: basal ganglia thalamus pattern, PVL: periventricular leukomalacia, B: bilateral, N: normal spectrum, Y: spectrum representing HIE, MDI: Mental Developmental Index, PDI: Psychomotor Developmental Index).
